# Numerical investigation structural protection measures for riverbank due to flood flow-driven damage

**DOI:** 10.1371/journal.pone.0354963

**Published:** 2026-08-04

**Authors:** Thu Hien Le

**Affiliations:** Faculty of Water Resources Engineering, Thuyloi University, Kim Lien, Hanoi, Vietnam; Van Lang University: Truong Dai hoc Van Lang, VIET NAM

## Abstract

A three-dimensional Computational Fluid Dynamics (CFD) model was employed to investigate the impact of structural protection measures on riverbanks subjected to flood flow-driven damage. Three structural approaches are applied to a case study the downstream river of Cua Dat spillway: (i) three groups of submerged groynes with varying height ratios *R* = 1.04, 2.13 and 3.14 of flow depth (*d*_*o*_) to groyne height (*h*_*g*_), (ii) detention zones and (iii) concrete lining. Several hydraulic characteristics at near-bank flow, such as *Q*_*vortex*_, near-bank velocity (*V*_*bank*_) and impact load (*F*) acting on bank protection slabs. Groups of submerged groynes redirect high-velocity flow toward the mainstream, which may jeopardize the integrity of bridge piers. The lower-submergence scenario (*R* = 1.04) generates maximum impact forces on the upper slab layer approximately 3.5 times greater than those induced by no protection measure. Results indicate that excavating natural detention zones along the left riverbank, in combination with concrete lining on the right riverbank, effectively reduces flood risk not only at structurally vulnerable zones but also at the bridge. These findings provide practical insights into the hydraulic performance of structural measures for riverbank stabilization in high-risk spillway environments.

## 1. Introduction

Floods continue to cause severe and persistent socio-economic impacts worldwide, particularly in low- and middle-income countries where high exposure often coincides with poverty and limited adaptive capacity. Approximately 23% of the global population is exposed to 1-in-100-year flood events, with nearly 89% of this exposed population residing in low- and middle-income countries [1]. Vietnam ranks among the top 10 countries in terms of absolute exposure, with nearly half of its population living in flood-prone areas, especially along major river systems [2].

Pluvial and fluvial flooding have been widely investigated in previous studies [3,4]. However, riverbank instability induced by high-velocity spillway releases from large reservoirs remains comparatively underexplored. Although reservoirs are primarily designed to attenuate downstream flood risk, emergency releases through spillways and bottom outlets can generate highly energetic flows capable of imposing severe erosive and hydrodynamic forces on downstream riverbanks. Recent flood events in Vietnam, including those associated with the Son La and Cua Dat reservoirs, have demonstrated that large spillway discharges may cause substantial riverbank damage even where structural protection measures are already in place. Riverbank deformation results from complex interactions among hydrodynamic forcing, bank-material properties, vegetation, seepage, pore-water pressure fluctuations, overbank flow, and subaerial processes [5]. Recent studies have improved the understanding of bank-failure mechanisms under variable hydraulic conditions; for example, Hao et al. (2023) examined the collapse process of dual-structure vegetated riverbanks under fluctuating water levels [6]. Nevertheless, the specific interaction between extreme spillway-induced high-velocity flows and protected riverbanks downstream of large reservoirs has received limited attention. In such environments, concentrated near-bank velocities and coherent vortical structures can generate substantial hydrodynamic loads, potentially leading to instability, displacement, or breakage of concrete protection slabs.

A wide range of hard engineering measures, commonly referred to as *gray infrastructure*, has been adopted to mitigate riverbank erosion. These measures include riprap revetments, concrete linings, groynes, spur dikes, embankments, levees, and detention areas [4,7]. Riprap revetments are generally suitable for low- to moderate-velocity conditions, whereas concrete linings are often preferred in zones subjected to stronger hydraulic loading. Groynes are widely used to deflect high-velocity currents away from vulnerable banks; however, their hydraulic performance is strongly governed by geometry, orientation, submergence ratio, and local flow conditions [8]. Previous studies have shown that turbulence structures and secondary flows around groynes may both stabilize and destabilize riverbanks by redistributing momentum, modifying sediment transport, and inducing local scour [9]. Laboratory investigations have demonstrated the influence of groyne orientation on sediment deposition and bed stabilization [10,11] while recent numerical studies have quantified their role in reducing local scour [12,13]. Despite these advances, the influence of relative submerged-groyne height on near-bank velocity and hydrodynamic loading on riverbank protection slabs has not been adequately addressed. In addition to groynes and detention zones are important structural and semi-structural measures for flood-risk mitigation. By temporarily storing floodwater and expanding the effective flow area, these zones can reduce local flow momentum, attenuate downstream discharge peaks, and promote controlled inundation in less vulnerable areas [14,15]. However, their combined hydraulic effect with hard bank-protection structures under high-velocity spillway releases remains insufficiently quantified.

Both physical and numerical approaches have been used to investigate riverbank protection and flow–structure interaction. Physical models remain valuable for controlled laboratory experiments and direct observation of hydraulic processes [16,17]. Nevertheless, they are often constrained by scale effects, measurement density, and the difficulty of reproducing complex prototype geometries. Three-dimensional computational fluid dynamics (CFD) has therefore become an effective tool for resolving flow structures around bank-protection works, including velocity fields, pressure distributions, turbulence patterns, shear stresses, and vortex development [18]. Such capabilities make CFD particularly suitable for assessing alternative rehabilitation measures in complex downstream reaches affected by extreme spillway discharges. However, few studies have applied 3D CFD to clarify the hydraulic mechanisms by which spillway-induced high-velocity flows interact with protected riverbanks and to quantify the resulting loads on protection slabs.

In Vietnam, structural failures along riverbanks downstream of spillways have still occurred during large flood releases despite the prior installation of protective works. This study addresses this knowledge gap by numerically investigating the hydraulic effectiveness of alternative riverbank-protection measures downstream of the Cua Dat reservoir under an extreme spillway-discharge condition. A 3D CFD model is employed to analyze near-bank hydraulic parameters, including velocity distribution, vortex intensity, water level variation, and hydrodynamic force acting on protection slabs. The specific objectives are to: (i) identify the hydraulic mechanisms responsible for flow-induced riverbank damage; (ii) evaluate the influence of submerged-groyne height on near-bank flow and slab loading; and (iii) compare the performance of groynes, detention-zone excavation, and concrete lining as potential rehabilitation strategies. The findings provide practical insights for designing more effective riverbank-protection systems in high-risk downstream environments subjected to high-velocity reservoir releases. Although derived from the Cua Dat case, the identified relationships between submerged-groyne height, momentum redistribution, vortex development, and detention-zone-induced energy dissipation are governed by fundamental hydraulic processes rather than local boundary conditions alone. Therefore, the proposed rehabilitation concepts and associated hydraulic responses are expected to be applicable to other spillway downstream river experiencing high-velocity flood releases, providing transferable guidance for riverbank stabilization in reservoir-regulated rivers.

## 2. Methodology and case study

### 2.1. Description of case study

**Fig 1:**
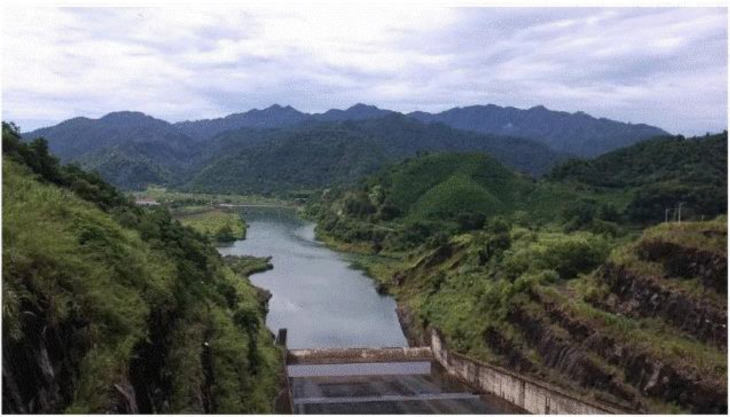
Downstream river of the case-study spillway (photograph by the author).

Downstream river reach of large reservoir Cua Dat (Vietnam) is often exposed to high-velocity flows during extreme flood events. Once the reservoir water level exceeds the normal operating threshold, substantial discharges must be released through spillways and bottom outlets to ensure dam safety [19]. During the 2007 flood event, severe erosion at the left bridge abutment resulted in the loss of approximately 35 m of the left riverbank and disrupted local transportation. A decade later, a peak spillway discharge of about 3,700 m³/s was released from the Cua Dat reservoir to ensure dam safety. Despite previous rehabilitation efforts, the bridge abutment failed again, and nearly 360 m of the upstream left bank was destroyed, indicating the limited effectiveness of the existing protection measures. Although no damage was observed on the right bank during previous floods, the steep bank geometry and the additional flow supplied by the hydropower canal produce relatively high near-bank velocities. The presence of a roadway along the right bank further increases the consequences of potential bank failure. Therefore, a comprehensive assessment is needed to identify effective protection measures capable of mitigating flood-induced riverbank damage and safeguarding critical infrastructure (Fig. 1).

### 2.2. Numerical model

The Navier–Stokes equations are a set of partial differential equations that describe the motion of fluid substances such as liquids and gases. These equations arise from applying Newton’s second law of motion to fluid motion, along with the assumption that the fluid stress is the sum of a diffusing viscous term and a pressure term.

The equations describing an incompressible fluid in three dimensions can be expressed in a general form as follows:


$$\begin{array}{c} \nabla .𝐮=0 \end{array}$$ (1)

$$\begin{array}{c} \rho \left(\frac{\partial 𝐮}{\partial t}+𝐮.\nabla 𝐮\right)=-\nabla p+\mu \nabla^{\text{2}}𝐮+\rho 𝐠 \end{array}$$ (2)

Where: **u**: velocity vector; *t*: time; *ρ*:fluid density; *p*: pressure; *μ*: dynamic viscosity; ∇^2^: Laplacian operator; **g**: gravitational acceleration vector.

Eq. (1) articulates the principle of mass conservation, indicating that the divergence of the velocity field is implicated. Eq. (2) relates to the principle of momentum conservation.

Free-surface and turbulent flow features were simulated using the Volume of Fluid (VOF) method coupled with Reynolds-averaged Navier–Stokes (RANS) equations. The VOF formulation tracks the evolution of the air–water interface by solving an additional transport equation for the fluid volume fraction, enabling accurate representation of complex free-surface behavior. Turbulence effects were represented through time-averaged flow equations, which provide a computationally efficient framework for reproducing the dominant characteristics of highly turbulent hydraulic flows without explicitly resolving all turbulent scales.

Numerical simulations were carried out using Flow Hydro, a 3D CFD package developed for hydraulic and environmental applications. The software incorporates advanced free-surface algorithms and specialized modules for simulating a wide range of hydraulic phenomena. In the present study, the Renormalization Group (RNG) turbulence closure, belonging to the RANS family, was adopted because of its proven capability to reproduce complex flow patterns around hydraulic structures and under rapidly varying flow conditions.

### 2.3. Computational domain, boundary condition and validation

**Fig 2:**
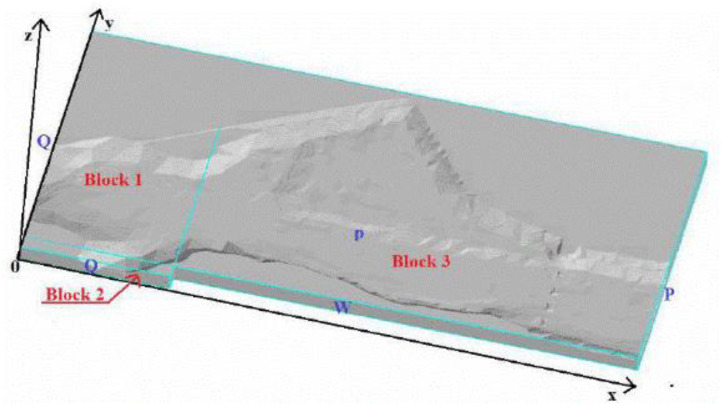
Schematic of computational domain and boundary conditions.

The 3D computational domain encompasses a 900 m length downstream of the spillway, with bathymetric elevations ranging from 26.26 m to 32.54 m. The domain is segmented into three distinct blocks. The block 1 is located post-spillway, the block 2 encompasses the lateral hydropower canal, and the final block comprises the remainder of the domain (Fig 2).

In this study, a steady flow condition was adopted to simulate the hydraulic characteristics within the 3D domain. Accordingly, constant values were prescribed at all boundary conditions (Fig 2). The maximum discharge released from the reservoir spillway, was imposed at the X_min_ boundary of Block 1. In addition, the Y boundary of block 2 represents a constant discharge emanating from the hydropower house. The channel’s side walls and bottom were designated as walls (W). The upper boundary Z of three blocks was designated as a specific pressure, with a fluid fraction established at 0.0 to represent the free surface flow condition (Table 1).

**Table 1:** Boundary conditions of computational domain.

Blocks	X		Y		Z	
**X**_**min**_	**X**_**max**_	**Y**_**min**_	**Y**_**max**_	**Z**_**min**_	**Z**_**max**_
Block1	Q, z	S	S	W	W	P (fluid fraction = 0)
Block2	S	S	Q (*Q* = 105 m^3^/s, *z* = 37.2 m)	S	W	P (fluid fraction = 0)
Block3	S	P	S	W	W	P (fluid fraction = 0)

Water level time series at the downstream boundary (X_max_ of block 3) were obtained from a two-dimensional MIKE 21 model applied to a larger domain, with boundary conditions derived from hydrological data and Cua Dat spillway operation schedules. Model performance was validated using water level records from an automated monitoring station at Cua Dat bridge during the 2017 flood event. However, due to the lack of field measurements, near-bank velocities and vortex structures were not directly validated and are therefore interpreted as physically based numerical predictions. The resulting stage hydrograph was used to define the downstream boundary of the 3D domain, while maximum discharge and corresponding water levels at X_min_ (block 1) and X_max_ (block 3) were specified as boundary conditions. The 3D model was validated against observed water levels at the monitoring station, and mesh sensitivity analysis was performed to determine the optimal grid resolution (Table 2). Table 2 shows that mesh refinement improved model accuracy, reducing the relative error from 1.45% for the 1.25 m grid to 0.74% for the 1.0 m grid. Further refinement to 0.8 m yielded only a marginal improvement (0.72%) while substantially increasing computational cost and file size. Therefore, the 1.0 m mesh was selected for subsequent simulations because it provides a good balance between accuracy and computational efficiency.

**Table 2:** Mesh sensitivity analysis.

Simulation case	Grid size (m)	Simulation time (s)	Time consuming (h)	File size (GB)	Z_obs_ (m)	Z_sim_ (m)	Relative error (%)
2017 flood event (Q = 4009 m^3^/s)	1.25	1000	7	15	32.41	31.94	1.45
	1.0	1000	20	60	32.41	32.65	0.74
	0.8	1000	35	132	32.41	32.55	0.72

Subsequent scenario simulations were conducted following model validation, using the design flood with a frequency of 0.6% as input. In these simulations, the maximum discharge at the upstream boundary of 3D domain was imposed as 3401.6 m³/s, while corresponding water levels of 37.21 m and 37.11 m were imposed at the upstream and downstream boundaries, respectively.

### 2.4 Bank protection scenarios

**Fig 3:**
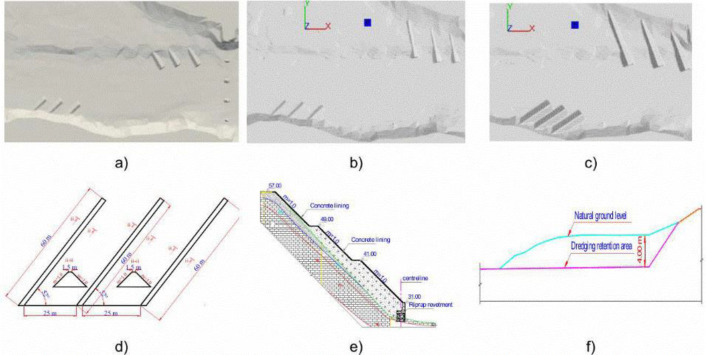
Configuration of remedial structural approaches: a) L + R; b) L1 + R1; c) L2 + R2; d) Dimensions of groynes group; e) R3; f) R4.

The riverbanks and channel foundation are composed of highly cohesive soils with considerable shear strength and resistance to scour; therefore, bed and bank erosion are assumed to have a negligible influence on the present analysis. This study evaluates three riverbank protection measures, namely submerged groyne groups, concrete bank lining, and dredging of the detention zone, and quantifies their effects on the hydraulic characteristics of high-velocity flows generated by large spillway releases. The objectives are twofold: (i) to assess the effectiveness of these measures in reducing near-bank flow velocity, and (ii) to determine their influence on the hydrodynamic loads acting on bank protection structures (Table 3). Groynes are primarily intended to regulate channel alignment, reduce flow concentration near vulnerable sections, and mitigate the impact of high-velocity currents on riverbanks. In this study, groups of three submerged groynes are installed at critical locations along both banks to alter the trajectory of high-energy flows. The structures are designed as attracting spurs, oriented downstream at an angle of 45°–60°, thereby deflecting the flow toward the groyne itself and reducing the hydraulic attack on the opposite bank. To investigate the effect of groyne height, three crest elevation scenarios are considered. In Case 1, groynes L and R have crest elevations of 30.0 m; in Case 2, groynes L1 and R1 are raised to 31.0 m; and in Case 3, groynes L2 and R2 have crest elevations of 35.0 m (Fig 3a–3d). These configurations provide the basis for evaluating the influence of submerged groyne height on near-bank velocities and the hydrodynamic forces acting on bank protection slabs.

**Table 3:** Simulation bank protection scenarios.

Scenarios	Left bank	Scenarios	Right bank
L0	Real case study	R0	Real case study
L	Groynes with top elevation + 30.0m	R	Groynes with top elevation + 30.0m
L1	Groynes with top elevation + 31.0m	R1	Groynes with top elevation + 31.0m
L2	Groynes with top elevation + 35.0m	R2	Groynes with top elevation + 35.0m
L3	Dredging natural detention zone	R3	Slope concrete lining with two tiers

Concrete lining is one of the most widely used riverbank protection measures because of its ability to prevent erosion and improve hydraulic efficiency. This approach is particularly suitable for reaches exposed to frequent flash floods. The smooth concrete surface reduces flow resistance, increases channel conveyance capacity, and minimizes sediment deposition. In addition, concrete lining can be combined with retaining walls to further enhance overall bank stability (Fig 3e).

Furthermore, a detention zone is a natural or engineered storage area designed to temporarily retain excess water during high-flow events. By attenuating peak discharges and reducing flow velocity, it contributes to flood mitigation while limiting erosion and hydraulic damage to the channel. In the present study, the detention zone is deepened by dredging to a maximum depth of 4.0 m (Fig 3f). The increased storage capacity and enlarged flow cross-section are expected to dissipate flow energy and reduce the intensity of high-velocity currents near the riverbanks.

## 3. Results and discussions

### 3.1. Mesh sensitivity analysis

The processing duration and mesh quality significantly influence the computing expenses and precision of modeling outcomes, respectively (Table 4). All simulations are executed on the Intel® Xeon CPU E5-2686 v4 operating at 2.3GHz. The optimal grid size must fulfill the requirements of operating time, file size, and the Grid Convergence Index (GCI).

**Table 4:** The processing time and total cells.

ID	Cell size (m)	Time (h)	Result file size (GB)	Total Cells
1	1.00	16h25’32s	24	11,320,678
2	1.20	9h10’04s	15	6,806,481
3	1.44	3h41’28s	9	3,994,080
4	1.73	1h32’56s	6	2,389,668

**Fig 4:**
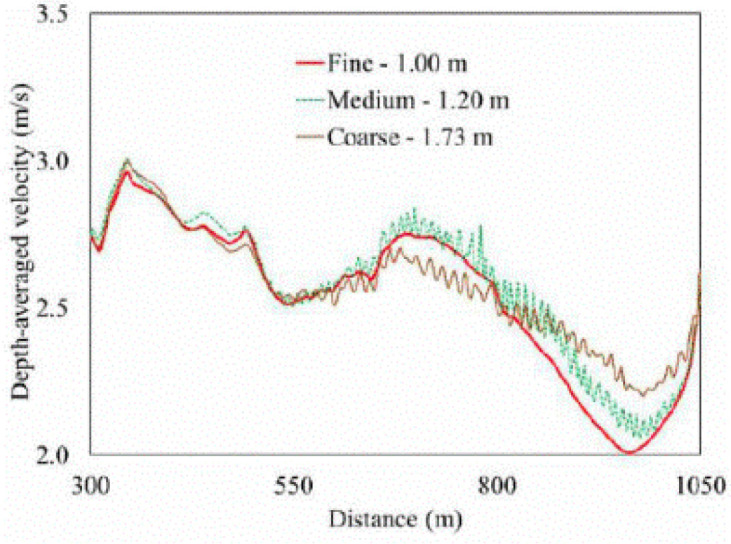
Depth-averaged velocity distribution along centerline of the river segment.

Fig 4 compares the depth-averaged velocity profiles along the centerline of the study reach obtained using three mesh resolutions: 1.73 m, 1.2 m, and 1.0 m. In the downstream half of the reach, simulations with the coarser meshes (1.73 m and 1.2 m) exhibit pronounced oscillations, whereas the 1.0 m mesh yields a considerably smoother profile. This behavior highlights the sensitivity of the numerical solution to mesh resolution. Coarse meshes may introduce numerical dispersion, especially in regions with strong flow gradients and boundary layers, whereas finer meshes reduce these spurious oscillations and improve solution accuracy. Table 5 summarizes the GCI values of the depth-averaged velocity at five representative cross sections (x = 400.5, 490.5, 705.5, 1055.5, and 1180.5 m) for three mesh resolutions of 1.44 m, 1.2 m, and 1.0 m. At all cross sections, the GCI_32_ values are consistently larger than the corresponding GCI_21_ values, indicating that the numerical solution approaches grid independence and that further mesh refinement would yield only marginal improvements. Moreover, the ratios GCI_32_/(r^p^ GCI_21_) are close to unity, confirming that the solutions lie within the asymptotic range of convergence. Therefore, a grid spacing of 1.0 m was deemed sufficient to achieve reliable numerical accuracy and was adopted for all simulations. With this mesh resolution, each computational scenario consists of approximately 11.3 million cells.

**Table 5:** Mesh convergence analysis.

N^0^	Cell size (m)	X = 400.5 m		X = 490.5 m		X = 705.5 m		1055.5 m		X = 1180.5 m	
**V** (m/s)	**GCI** (%)	**V** (m/s)	**GCI** (%)	**V** (m/s)	**GCI** (%)	**V** (m/s)	**GCI** (%)	**V** (m/s)	**GCI** (%)
1	1.00	2.8254		2.762		2.741		2.605		2.516	
2	1.20	2.8255	1.8.10^-6^	2.779	0.562	2.761	0.172	2.596	0.032	2.431	4.72
3	1.44	2.8256	1.5.10^-3^	2.736	1.356	2.623	1.099	2.486	0.430	2.270	9.25
GCI_32_/r^p^GCI_21_		1.000		0.994		0.993		1.003		1.035	

### 3.2. Numerical assessment of the 0.6% flood event

**Fig 5:**
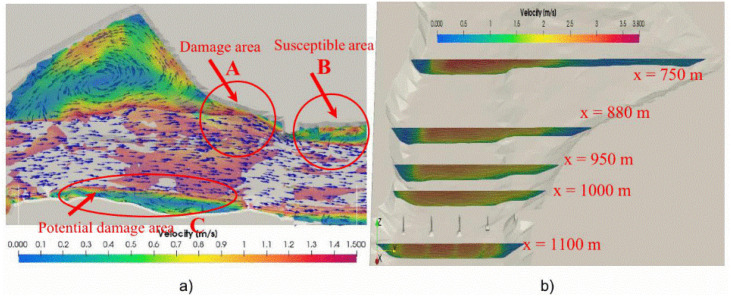
Velocity distributions: a) Threshold velocity magnitude within the range of 0 to 1.5 m/s; b) Velocity magnitude distributions in different cross sections.

Fig 5a shows that depth-averaged velocities along both riverbanks and the channel bed upstream of the bridge generally range from 0 to 1.5 m/s. However, high-velocity currents are concentrated and directed toward the vulnerable left-bank section (Region A), which explains the failure of the concrete bank protection slabs observed in the field. In Region B, a localized high-velocity zone develops adjacent to the embankment, accompanied by pronounced vortical structures that may increase the risk of erosion and structural damage. In addition, the right bank is characterized by a steep, unprotected slope, where velocities of up to 1.5 m/s and localized vortex formation occur within a narrow near-bank zone (Region C). These hydraulic conditions indicate that protective measures are required to ensure the stability of the right bank and to safeguard the roadway located above it.

In addition, post-processing in FlowHydro employs Line Integral Convolution (LIC), a flow visualization technique that generates textured images to illustrate the direction and structure of vector fields such as velocity and vorticity. Fig 5b presents the LIC-based velocity distributions at several representative cross-sections. In general, the high-velocity core is concentrated near the center of the channel. At x = 880 m, vortex structures are observed near the left side of the channel bed. At x = 950 m and 1000 m, the velocity near the left bank exhibits strong vertical variations, increasing from approximately 1.5 m/s close to the bed to 2.0–2.5 m/s near the water surface. Downstream of the bridge (x = 1100 m), a distinct vortex develops near the bottom left corner of the cross-section. Field observations have also revealed signs of deterioration along the left bank adjacent to the bridge pier (Region B), suggesting that this section remains susceptible to future failure. Therefore, a detailed understanding of the flow characteristics and potential failure mechanisms near the riverbanks is essential for identifying effective rehabilitation strategies. Accordingly, three structural protection measures are proposed to mitigate the adverse effects of high-velocity flow interaction with the riverbanks.

### 3.3. Numerical results of protection measure scenarios

Under high-velocity flood conditions, the coupled interaction between near-bank hydraulics and the geotechnical resistance of bank materials governs the transition from localized erosion to large-scale bank instability. Such failures can subsequently modify channel morphology and exacerbate flood risks in downstream reaches.

#### 3.3.1. The influence of protection measures on hydraulic characteristics in spillway downstream river.

**Fig 6:**
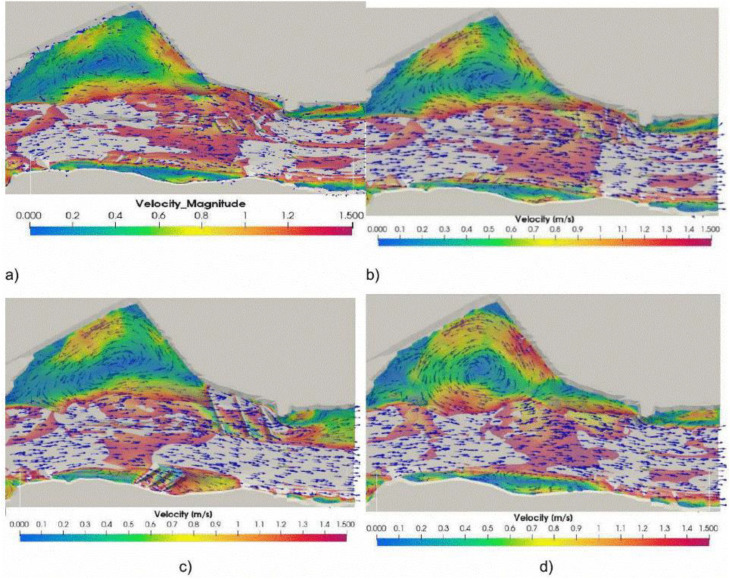
Threshold velocity magnitude in the range of (0-1.5) m/s of 4 protection measures: a) L + R; b) L1 + R1; c) L2 + R2; d) L3 + R3.

The deployment of groups of submerged groynes along both riverbanks effectively redirects the high-velocity mainstream away from critical zones. Compared with the baseline scenario (L0 + R0), flow velocity in the lower water layer adjacent to the riverbank toe is reduced, whereas velocity downstream of the bridge increases (Figs 6a–c). However, for scenarios with relatively high groynes (L1 + R1), the reduction in near-bank velocity along either bank remains limited. In contrast, the L2 + R2 configuration, with a crest elevation of 35 m, substantially decreases velocity in the lower water layer while increasing it in the upper layer within region A and rapidly reducing it within region B (Fig 6c). Nevertheless, these groyne configurations are ineffective in protecting the right bank, as the low-velocity zone (0–1.5 m/s) does not expand relative to the unprotected L0 + R0 scenario (Fig 5a). The downstream reach of the Cua Dat spillway naturally contains a detention area that becomes inundated only during flood events. This area attenuates flood peaks by temporarily storing excess runoff, thereby reducing flow velocity and mitigating erosion. Excavation and dredging associated with the L3 scenario significantly modify the hydraulic characteristics of the flood flow. As shown in Fig 6d, the extent of the recirculation and stagnation zone within the detention area increases markedly, effectively diverting high-velocity flow away from the damaged section and reducing velocity magnitude in region A. In addition, concrete lining installed along the right bank improves bank stability and provides further protection against erosion.

Table 6 summarizes the maximum hydraulic parameters obtained from four simulation scenarios at cross-section x = 1034.5 m, immediately upstream of the bridge. The evaluated parameters include pressure head (*p/γ*), flow depth (*h*), water surface elevation (*z*), depth-averaged velocity (*V*), Froude number (*Fr*), and the Q-criterion (*Q*_*vortex*_) used to characterize vortex structures. The Q_vortex_ parameter quantifies the balance between rotational motion and strain in the flow field and identifies regions where vorticity dominates over deformation. Positive Q_vortex_ values indicate the presence of coherent swirling motions and stronger turbulent structures. Compared with the baseline case without protective measures (L0 + R0), the L2 + R2 configuration produces a slightly lower water level in front of the bridge. However, the depth-averaged velocity increases markedly from 2.62 to 3.57 m/s, resulting in the highest Froude number among all scenarios. Furthermore, the intensity of vortical structures is substantially enhanced. The maximum *Q*_*vortex*_ values associated with the groyne configurations reach 3.20 and 4.89 1/s ⁻ ², respectively, which are considerably higher than the corresponding value of 1.29 1/s ⁻ ² obtained for the L0 + R0 case. These results indicate that, although submerged groynes effectively reduce near-bank velocities, they also accelerate the main flow and intensify vortex structures in front of the bridge, potentially increasing local scour and hydraulic instability.

**Table 6:** Maximum hydraulic characteristics in front of bridge.

Simulation scenarios	*p/γ*(m)	*h* (m)	*z* (m)	*V* (m/s)	*Fr* (-)	*Q*_*votex*_ (1/s^2^)
L0 + R0	10.69	11.10	37.96	2.62	0.42	1.29
L + R	10.60	11.07	37.96	2.70	0.52	2.55
L1 + R1	10.61	11.00	37.97	2.92	0.74	3.20
L2 + R2	10.69	11.06	37.29	3.57	0.84	4.89
L3 + R3	10.67	11.05	37.84	2.78	0.39	0.95

**Fig 7:**
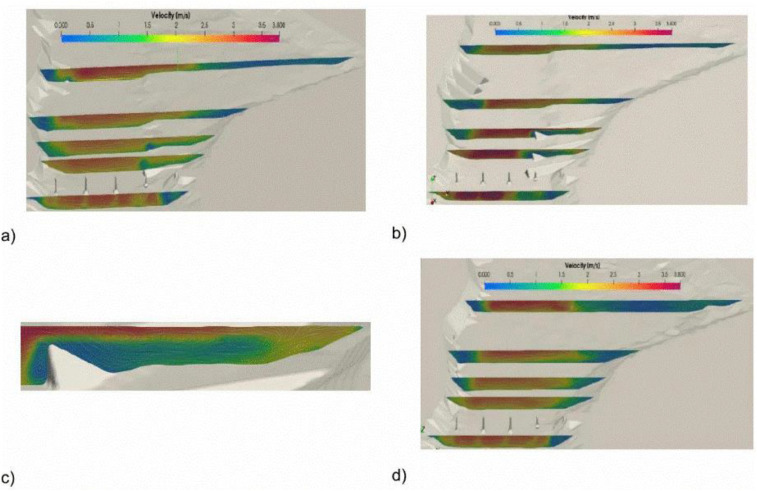
Velocity magnitude distributions in different crosssections of two submerged groyne scenarios: a) L1 + R; b) L2 + R2; c) Vortex formation in the vicinity of a groyne L2; d) L3 + R3.

Figs 7a and 7b illustrate the effects of the submerged groynes and detention-zone rehabilitation measures on the velocity distributions at several cross-sections. In the L2 + R2 scenario, the increased groyne height significantly alters the flow pattern by redirecting the high-velocity mainstream away from the protected bank. However, the resulting velocity magnitude in the main channel remains higher than that observed in the L3 + R3 scenario (Fig 7d). In particular, a strong vortex develops near the groyne toe, as evidenced by the Q-criterion contours (Fig 7c). Above the groyne field, a high-velocity layer forms due to flow contraction and overtopping, whereas a low-velocity region is established close to the riverbed and within the groyne field. This sheltered zone reduces near-bed shear stresses and consequently diminishes the potential for local scour around the bank toe (Fig 7c).

**Fig 8:**
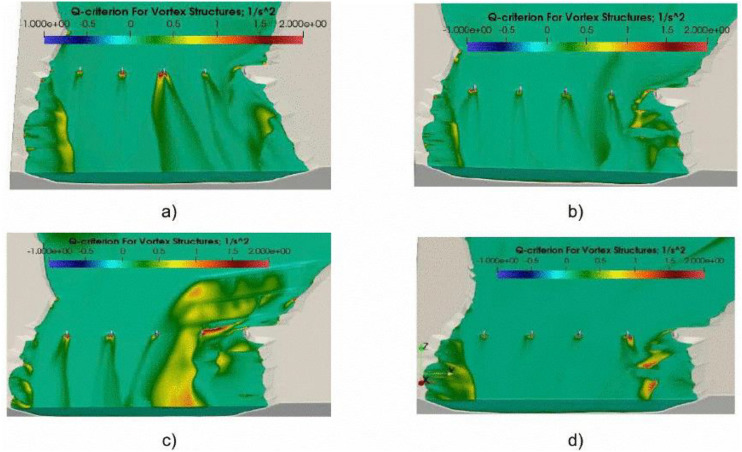
Q-criterion for vortex structures on the surface flow: a) L+ + R; b) L1+ + R1; c) L2+ + R2; d) L3+ + R3.

Fig 8 presents the Q-criterion visualization of the surface flow, highlighting coherent vortex structures in which rotational motion dominates over strain. In the L2 + R2 scenario, vortex intensity is substantially greater than in the L1 + R1 and L3 + R3 cases, demonstrating the strong influence of increased groyne height on surface circulation. These vortical structures dissipate flow energy and reduce near-bank velocities, thereby providing some protection against bank erosion. In addition, detached eddies promote localized sediment deposition and contribute to flow redistribution. However, the persistence of strong vortices near the groyne toes indicates a heightened risk of local scour.

Furthermore, the installation of groynes modifies channel hydraulics by contracting the effective flow area and accelerating the mainstream upstream and downstream of the structures. Although flow velocity within the lower layer of the groyne field is reduced, several adverse effects are observed. These include the transfer of erosive forces to adjacent reaches, increased mainstream velocities downstream of the groynes, and the generation of turbulence-induced vortices that intensify bed shear stresses and enhance riverbed scour. Consequently, while submerged groynes provide localized protection, their hydraulic impacts may inadvertently shift erosion problems to other parts of the channel.

#### 3.3.2. Quantitative the impact of group submerged groyne height and detention zone on near-bank flow.

Considering near-bank flow within zone A, this area witnessed the damage of bank protection slabs during two flood events in the past (Fig 2). The hydrodynamic force is calculated by Eq. (3).


$$\begin{array}{c} F_{bank}=\frac{\text{1}}{\text{2}}\rho C_{d}h_{s}V_{bank}^{\text{2}} \end{array}$$ (3)

Where: *ρ*: is density of water; *C*_*d*_: drag coefficient, riverbank protection design, guidelines of USACE EM 1110-2-1601 often recommend *C*_*d*_ = 1.0–1.2 for vertical slabs directly facing the flow; *h*_*s*_: the height of protection bank slab; *V*_*bank*_: near bank velocity, which is estimated by the 3D CFD model.

**Fig 9:**
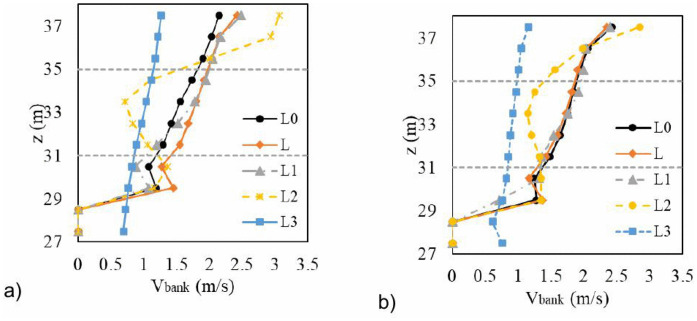
Near-bank velocity distribution profiles at two points within zone A: a) (950.5 m; 580. 5 m) and b) (974.5 m; 585.5 m).

Three groups of submerged groynes with varying degrees of submergence were examined. The relative submergence ratio, defined as the ratio of flow depth (*d*_*0*_) to groyne height (*h*_*g*_), *R = d*_*0*_*/h*_*g*_, was equal to 1.04, 2.13, and 3.14 for the L2 + R2, L1 + R1, and L + R scenarios, respectively. Fig 9 illustrates the influence of groyne height on near-bank velocity distributions in zone A for five simulation cases. The L2 + R2 scenario, characterized by the smallest submergence ratio (*R* = 1.04), reduces near-bed velocity to values below 1 m/s at elevations lower than +35 m, providing the greatest degree of protection among all cases. However, flow acceleration above the groyne crest produces maximum velocities of 3.07 and 2.85 m/s at elevations exceeding +35 m, indicating that the groynes locally intensify flow momentum and hydraulic forces in the upper water layer. By contrast, the L1 + R1 (*R* = 2.13) and L + R (*R* = 3.14) scenarios produce only minor changes in the velocity profile compared with the unprotected case (L0 + R0), suggesting that highly submerged groynes are less effective in modifying the flow field. These results indicate that groyne geometry and submergence must be carefully optimized to balance bank protection with undesirable increases in mainstream velocity. In comparison, the detention-zone excavation scenario (L3 + R3) provides a more uniform reduction in velocity throughout the water column. Velocity variations along the vertical direction are relatively small, and surface velocities are approximately half those observed in the groyne scenarios (Fig 9). This finding demonstrates that the detention zone dissipates flood energy more effectively and reduces near-bank hydraulic loading without causing significant flow acceleration in the upper layers.

**Fig 10:**
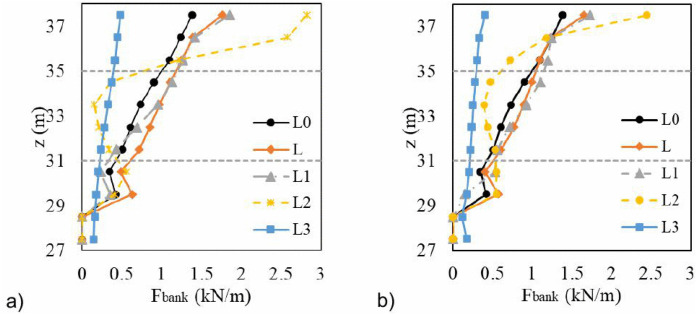
Hydrodynamic force distribution profiles at two points within zone A: a) (950.5 m; 580. 5 m) and b) (974.5 m; 585.5 m).

Furthermore, to quantify the hydraulic loading exerted by near-bank flow on the bank protection system, Eq. (3) was applied to a representative protection slab with a height of 0.5 m. Fig 10 illustrates the vertical distribution of the resulting impact force, (*F*_*bank*_). All groyne configurations produce larger impact forces on the upper portion of the protection slab than the unprotected case (L0 + R0). In particular, the L2 + R2 configuration substantially intensifies the hydraulic loading, with peak values nearly twice those obtained in the L0 + R0 scenario and approximately an order of magnitude greater than those in the L3 + R3 scenario. These results indicate that, although submerged groynes effectively reduce near-bed velocities, they redirect high-momentum flow toward the upper water layer, thereby increasing the hydraulic forces acting on bank protection structures.

Analysis of the maximum values of several hydraulic parameters in the vulnerable near-bank region A, including *p/γ*, *h*, *z*, *F*_*r*_, *Q*_*vortex*_, near-bank velocity (*V*_*bank*_), and hydraulic impact force (*F*_*bank*_), indicates that the presence of protective structures substantially reduces the water surface elevation compared with the unprotected scenario, as summarized in Table 7. Among the three groyne configurations, decreasing the relative submergence ratio *R* leads to pronounced increases in *Q*_*vortex*_, *V*_*bank*_, and *F*_*bank*_, although the corresponding Froude number tends to decrease. In particular, the L2 + R2 configuration (*R* = 1.04) generates considerably larger values of these parameters, implying intensified vortex activity, higher near-bank velocities, and greater hydraulic loading, which may increase the risk of bank instability and structural damage.

**Table 7:** Maximum hydraulic characteristics at vulnerable zone A.

Simulation scenarios	*p/γ*(m)	*h* (m)	*z* (m)	*Fr* (-)	*Q*_*votex*_ (1/s^2^)	*V*_*bank*_ (m/s)	*F*_*bank*_ (kN/m)
L0 + R0	7..79	7.4	39.34	0.4	0.91	2.49	1.85
L + R	7.76	7.52	37.3	0.3	1.77	2.52	1.9
L1 + R1	7.72	7.48	37.23	0.27	2.44	2.85	2.12
L2 + R2	7.87	7.58	37.4	0.76	4.92	4.05	6.61
L3 + R3	9.71	10.8	37.24	0.17	0.84	1.67	0.38

In contrast, the detention zone rehabilitation scenario (L3 + R3) markedly reduces *Q*_*vortex*_, *V*_*bank*_, and *F*_*bank*_, thereby alleviating hydraulic stresses and lowering the potential for erosion and failure of the bank protection system. Moreover, this solution may provide additional benefits during the dry season. As the discharge from the hydropower channel is diverted into the Chu River, water retained within the detention area can act as a buffer zone, weakening the interaction between the diverted flow and the left riverbank and consequently reducing long-term erosion potential.

## 4. Conclusions

This study employed a three-dimensional CFD model to investigate the hydraulic performance of three riverbank rehabilitation strategies under the 0.6% exceedance probability flood downstream of the Cua Dat spillway. The simulations successfully captured the complex hydraulic processes associated with high-velocity flows, including velocity distributions, vortex structures, and turbulent flow regions, demonstrating the capability of CFD as an effective tool for evaluating alternative protection measures.The numerical solution indicated that:

Groups of submerged groynes effectively redirect high-velocity flow away from the riverbanks toward the channel centerline. However, this flow contraction increases velocity and shear stresses in the mainstream, potentially threatening the stability of bridge piers. A lower relative submergence ratio (small *R = d*_*0*_*/h*_*g*_) provides the greatest reduction in near-bank velocity and hydraulic impact force near the bed, but simultaneously intensifies upper-layer flow, vortex structures, and turbulence compared with configurations having larger R values.

The detention-zone excavation approach effectively attenuates flood energy by temporarily storing excess floodwater, thereby reducing flow velocities both near the riverbanks and within the mainstream. In addition, vortex development in the vicinity of the banks remains weak, which lowers bed shear stresses and reduces the likelihood of local scour. Consequently, this approach provides a more balanced hydraulic response and minimizes the transfer of erosion hazards to adjacent channel reaches.

It should be noted that this study considered only hydrodynamic responses and loading. Sediment transport, local scour, and morphological evolution were not simulated because the riverbed and banks are highly cohesive. Therefore, the results should be interpreted as indicators of hydraulic loading and potential damage risk rather than actual morphological changes. Overall, near-bank hydraulic characteristics, particularly velocity and impact forces, are critical for evaluating riverbank protection measures. Although gray infrastructure remains essential in high-risk reaches, integrating nature-based approaches with conventional structures offers a more sustainable and resilient strategy for mitigating flood-induced riverbank damage.
